# The Effectiveness of Advanced Practice Nurses with Respect to Complex Chronic Wounds in the Management of Venous Ulcers

**DOI:** 10.3390/ijerph16245037

**Published:** 2019-12-11

**Authors:** Juan Francisco Jiménez-García, Gabriel Aguilera-Manrique, Josefina Arboledas-Bellón, María Gutiérrez-García, Francisco González-Jiménez, Nieves Lafuente-Robles, Laura Parra-Anguita, Francisco Pedro García-Fernández

**Affiliations:** 1 Advanced Practice Nurse in Complex Chronic Wound, Sanitary District Poniente of Almería, 04746 Venta del Viso, Spain; 2 Nursing Department, Dean of the Faculty of Health Sciences, University of Almería, 04120 Almería, Spain; gaguiler@ual.es; 3 Advanced Practice Nurse in Complex Chronic Wound, Sanitary District Jaén Northeast, 23400 Úbeda, Spain; josarbol@yahoo.es; 4 Advanced Practice Nurse in Complex Chronic Wound, Sanitary District Serrania of Malaga, 29400 Ronda, Spain; maria.gutierrez.garcia.sspa@juntadeandalucia.es; 5 Advanced Practice Nurse in Complex Chronic Wound, Sanitary District Metropolitano of Granada, 18016 Granada, Spain; pacomollimolli@gmail.com; 6 Director of the Comprehensive Care Plan for Andalusia, 41001 Sevilla, Spain; nieves.lafuente.sspa@juntadeandalucia.es; 7 Health Sciences Department, University of Jaen, 23071 Jaén, Spain; lparra@ujaen.es; 8 Nursing Department, Vice-Dean of the Faculty of Health Sciences, University of Jaén, 23071 Jaén, Spain; fpgarcia@ujaen.es

**Keywords:** venous ulcers, effectiveness, advanced practice nurse, complex chronic wounds

## Abstract

This study aims to evaluate the effectiveness of advanced practice nurses with respect to complex chronic wounds (APN-CCWs) in the care of patients with venous ulcers. A multicentric, quasi-experimental pre-post study was conducted without a control group in the sanitary management areas where the APN-CCW program is being piloted. The intervention consisted of a mass training of clinical nurses from the participating districts on the proper management of injuries and the use of compression therapy. The data were collected through a specifically constructed questionnaire with questions regarding descriptive variables of injuries and their treatment. A total of 643 professionals responded (response rate of 89.1%), attending to a total population of 707,814 inhabitants. An increase in multilayer bandage use by 15.67%, an increase in elastic bandage use by 13.24%, and a significant decrease in the referral of patients to consultation with hospital specialists was achieved, from 21.08% to 12.34%. The number of patients referred to the APNs was 13.25%, which implied a resolution rate of 94.08% of their injuries. In conclusion, the coordination by the APN-CCWs in patients with venous ulcers was effective in improving the continuity of care, in the optimization of resources, and in their care role.

## 1. Introduction

Venous ulcers (VU) negatively affect the quality of life of patients and represent an elevated cost both socially and in healthcare, because they are the most frequently seen ulcers in the lower limbs (70%–80%). They are also the most common in diagnosis, treatment, and prevention of relapses [1]. These ulcers underlie chronic venous insufficiency (CVI) with a common pathophysiological basis, such as chronic venous hypertension (VHT) with valvular insufficiency in the lower extremities [2]. It is estimated that venous ulcers affect 1%–2% of the world population. They are more prevalent in people older than the age of 65, directly interfering in their quality of life due to the chronic conditions related to the disease [3].

In a patient with a venous ulcer, compression therapy is the cornerstone to managing patients with venous ulceration of the lower limb and is considered to be the standard first-line clinical treatment. Compression can be achieved using several methods, including the use of a single component or layer (such as a compression stocking or bandage) or the use of multiple components or layers (different types of bandages or stockings and bandages used together) [4]. The effects of compression on the reduction of edema and pain and its ability to increase the healing rates of venous insufficiency lesions have been highlighted in the scientific literature through several systematic reviews [3,5,6,7,8], which have also highlighted its benefits with respect to venous ulcer recurrence. In these cases, in addition to effective compression therapy, it is important and essential to plan and coordinate the care and follow-up of these patients. As Bellmunt et al. say, “The problem of coordination has never occupied a privileged place on the agenda of healthcare authorities, although it is increasingly necessary due to the high prevalence of chronic diseases, comorbidity and the presence of new technologies; in short, the need to ensure continuity in care, since it is necessary to understand the coordination between levels as a synonym of care continuity” [2]. For this reason, the Public Health System of Andalusia (Spain) has developed and set in motion a program promoting the figure of the advanced practice nurse with respect to complex chronic wounds (APN-CCW). The advanced practice nurse undertakes different roles, such as researcher, clinician, change agent, teacher, consultant, and coordinator between levels of healthcare [9,10,11,12,13,14,15,16,17].

The general objective was to evaluate the effectiveness of the APN-CCW program in the care of venous ulcers. In addition, the specific objectives were to (1) determine the effect that the mass training conducted by the APN-CCW has on the care (management of injuries and use of compression therapy) of patients with venous lesions by clinical nurses and (2) understand the adequacy of the treatments of patients with venous ulcers.

## 2. Materials and Methods

### 2.1. Ethical Aspects

The study was approved by the Research Ethics Committee (48/2016), which confirmed that the study respected the ethical principles outlined in the Declaration of Helsinki in 2013 and other International Codes. All the participants signed an informed consent prior to their inclusion, ensuring data confidentiality and offering the choice to withdraw.

### 2.2. Design

A multicentric, quasi-experimental pre-post study with two measurements and without a control group was proposed, measuring the effectiveness of APN-CCWs in Andalusia in terms of preventive measures applied to patients with venous ulcers. The study took place in the sanitary districts (Sanitary District Poniente, Jaén Norte Sanitary District, Serranía de Ronda Sanitary District) in Andalusia where the APN-CCW program was piloted between September 2015 and October 2016. It was conducted in two consecutive phases. In the first phase, the pre-implantation situation of the APN-CCW program was analyzed by means of a self-administered questionnaire specifically constructed for data collection by the nurses of the aforementioned sanitary district. The population had a measured age of 78.16 years (CI 95% 77.07–79.24) of which 63.1% were women and the rest were men. The intervention was performed, consisting of mass training for all nurses on the use of preventive measures. Later, a comparative analysis was conducted after the implementation of the APN-CCW program one year later, using the same self-administered questionnaire.

### 2.3. The Study Unit

Population: The study consisted of all the nurses of the healthcare districts and healthcare management areas where the APN-CCW program was piloted.

Inclusion criteria: All the professionals who were in charge of patients with venous ulcers were included in their respective healthcare centers.

Exclusion criteria: Specialist nurses (mother–infant or mental health), case manager nurses, and nursing directors were excluded, because they are not in charge of patients with venous ulcers.

Type of sampling: Accidental or convenience sampling was used among the nurses who participated in the data collection in the two phases established in September 2015 and October 2016.

Sample size: This was a conceptual sample (including all clinical nurses of the participant districts), using the inclusion criteria previously proposed.

### 2.4. Intervention

The intervention consisted of a mass training of clinical nurses from the participating districts on the proper management of injuries and the use of compression therapy, with different types of elastic bandages, inelastic bandages, multilayers, and compression stockings.

The sessions were repeated until practically 100% of the nurses in each district were trained. The training was conducted by the APN-CCW in each district, with the same teaching content that was accredited by the Andalusian Training Accreditation Agency.

### 2.5. The Variables Analyzed

A descriptive analysis of the variables was performed. Quantitative variables are presented as mean and standard deviation and qualitative variables as frequencies and percentages.

Descriptive variables of primary care: The number of people in home care (PiHC), number of people bedridden, and number of people in consultations were considered.

Descriptive variables of the injuries and treatment: Age, gender, type of injury, origin, provenance, time of evolution, location, surface, depth, type of tissue, borders, exudate, presence of infection, increase in size, increase in pain, increase in exudate, presence of biofilm, presence of erythema, presence of exudate, stagnant wound, hyper granulation, presence of satellite injuries, presence of smell, presence of pallor, presence of reliable tissue, RESVECH score (expected results of the assessment and evolution in the healing of chronic wounds), presence of fistulas, perilesional skin condition, type of debridement, treatment of the wound bed, secondary dressing, perilesional skin treatment, use and type of bandages, pain, and adaptation of the treatment to the clinical practice guidelines of the Andalusian Health Service were considered.

### 2.6. The Method and Instruments for Data Collection

Data collection was conducted through an ad hoc questionnaire completed by all the nurses of the participant districts. The APN-CCW program was presented in clinical sessions in the different centers to train the nurses, and the collection was done in the same way. The final date of September 2015 was established for the first analysis cut and the end of October 2016 for the second. The documents and questionnaires (paper and computer format) for the data collection were created and followed by the data collection itself. The data were collected by each professional responsible for the patients and verified with them by each of the APN-CCWs, without interruption until collection was complete. Pain was measured using the visual analog scale [18] by the responsible nurse at the time of data collection.

### 2.7. Data Analysis

A descriptive analysis was conducted, calculating the frequency and percentage measures for the qualitative variables and measures of central tendency (mean) and dispersion (confidence interval or standard deviation) for the quantitative variables. The differences before and after the intervention (training of clinical nurses by the APN-CCW) were also analyzed.

## 3. Results

In the three districts, a total population of 707,814 inhabitants were served (30661 more than in 2015).

A total of 308 professionals responded to the questionnaire. The total number of patients in the PiHC program in the three sanitary districts was 6705. In this study, data corresponding to 5339 of those were obtained, representing the equivalent of 79.62%, of which 3533 (66.17%) were women and the remaining 1806 (33.83%) were men, which is 2.5% fewer women than in 2015. If we refer to ulcers in the lower limb, in the 6705 people included in the PiHC program and consultations, there were a total of 585 ulcers in lower limbs, which implies a prevalence of 8.72% (+1.81% more than in 2015). They had a total of 271 venous ulcers (46.33% of all ulcers in the lower limb), having increased +9.48% when compared with 2015.

An increase in the average age of patients suffering from venous ulcers was observed, and the high percentage of women who had this pathology exceeded 86% of the total, which was considered very significant. There was a significant decrease of over 16% in the percentage of women compared with men in 2016, as well as a decrease of over 3% of these injuries in primary care with a reduction of over one month regarding healing rates, which showed us that both training and preventive measures increased significantly.

A generalized improvement was also achieved regarding the type of tissue in the wound bed, the characteristics of the edges, and the amount of exudate; the data are shown in Table 1.

Similarly, a significant improvement was observed in the infection of venous ulcers and each of the clinical signs. The data are presented in Table 2.

Regarding the wound bed in venous ulcers, debridement is a fundamental part of good treatment. In the space of two years (2015 and 2016), it was observed that in more than 50% of cases no debridement was performed. Additionally, sharp debridement was used in less than 9% of the cases, which led us to believe that it is necessary to conduct specific training regarding the different types of debridement in order to show and demonstrate the advantages and benefits of each type of debridement to help and reduce the inflammatory phase in the healing process of venous ulcers.

**Figure 1:**
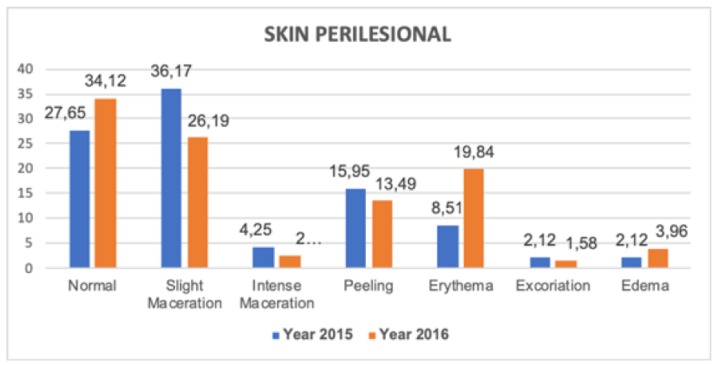
Characteristics of skin perilesional in venous ulcers.

**Figure 2:**
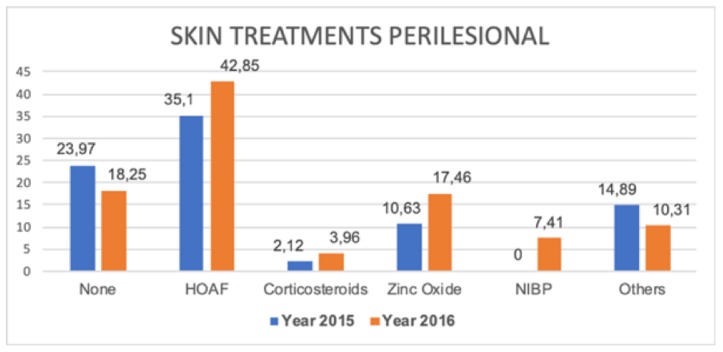
Treatment of perilesional skin in venous ulcers. Abbreviations: HOAF, hyper-oxygenated fatty acids; NIBP, non-irritating barrier products.

Regarding the state of the perilesional skin and its different treatments, in Figure 1 and Figure 2, less maceration and peeling and a higher percentage of normal skin in 2016 was observed, coinciding directly with evidence of greater use of hyper-oxygenated fatty acids, non-irritating barrier products, and ointments with zinc oxide.

With regards to the treatment applied, Table 3 shows the different types of bandages for patients with venous ulcers.

As can be seen, there were important improvements in the patients who were given elastic and multilayered bandages for the treatment of venous injuries, which reduced to less than one in 10 the percentage of patients who did not have any bandage on their injury. The increase in the use of the multilayer bandage (the most appropriate treatment) followed by the elastic bandage (the second most appropriate treatment) was especially striking, which highlights the importance of the training given by the APN-CCW program during this year.

There was also a significant change in terms of the pain reduction measured with a visual analog scale between 2015 and 2016. A reduction of about 19.20% in moderate pain with scores of 6–8 could be observed, as well as a reduction of about 1.10% in intense pain with scores of 9–10.

Similarly, an important decrease in the referral of patients to consultation with medical hospital specialists since the implementation of this program was also observed, going from 21.08% to 12.34%. At the same time, the number of patients referred to the APN-CCW was 13.25%, which shows that the existence of this program was important as a filter for the system; its capability of improving care for these patients was evident.

Of the 203 patients seen by the APN-CCW, only 12 were referred to a hospital specialist, which resulted in a resolution rate of 94.08% of the injuries referred to them.

## 4. Discussion

The results of our studies are part of a broader research project that is included in a doctoral thesis on the effectiveness of nurse care with respect to advanced chronic wound practice in Andalusia. Some of the research data show the effectiveness of the APN-CCW program on both pressure ulcers in primary care and leg venous ulcers. These data have already been published elsewhere [19].

The data presented here analyzed the effect of the APN-CCW program on venous etiology injuries. There was improvement in the optimization of the resources used to accomplish the objectives—progress in both the clinical trials and the decisions made. Furthermore, better results were achieved in terms of improving the rates of healing; reducing the severity and depth of the injuries; improving the condition of the wound bed, as well as its edges, exudate, infection, and pain; and acting on the causes of venous insufficiency with different compression systems [20,21,22].

As can be observed in the comparison of the venous ulcer characteristics results in 2015 and 2016, a significant improvement was achieved in terms of the reduction of necrotic and splotched tissue, the increase of the epithelization tissue at the level of the vein edges of the injuries, and the reduction of the state of thickened and damaged edges. In addition, in terms of the cleaning of the wounds, detritus, biofilm and exudate, and the leakage and saturation of the exudate were substantially improved, all of which are aspects that have been highlighted in the review by McLain et al. [23].

The fundamental key in the significant change obtained in the characteristics of venous ulcers was mainly attributed to the regulated training on the preparation of the wound bed and on the compression therapy against venous insufficiency and venous hypertension suffered by these patients, as well as the increase in patients after the health education conducted by the nurses. The improvement obtained in just one year of practically all the clinical signs of infection was clearly observed, as well as the significant improvement in terms of the elastic and multilayer compression therapy and the more than remarkable reduction of not using any type of bandage. These results are similar to those contributed by another series of authors [24,25,26], and those in turn coincided with other authors [7,27,28] in their systematic reviews. We would like to highlight the increase in the use of compression therapy during the post-test period, as we consider it fundamental for the treatment of these lesions and in the fundamental therapy for their healing.

Although the improvement in the results obtained was significant, there have also been points of improvement in our work that are related to the different types of debridements performed by nurses on patients with venous ulcers when they had necrotic or slough tissues in their ulcers (i.e., devitalized tissues and the limited use of compression stockings).

The non-performance of any type of debridement delays the healing process in venous ulcers and thus perpetuates the inflammatory phase. In addition, the percentages obtained from sharp debridement being the most effective in the removal of devitalized tissue, were very low compared with those of other authors in related studies [8,24,29]; therefore, a new program was added to the training that included the advantages of different types of debridement with their indications, contraindications, and side effects.

Although the data collected regarding sharp debridement performed by nurses were not as expected, it is considered a point of improvement, rather than a failure, for the next evaluation.

The results of the investigation also present a considerable limitation, as it has not been possible to compare them with a control group, because there is great variability in clinical practice, both with respect to the professionals and the patients themselves.

Until we gather more data, we will continue to believe that studies of patients with venous ulcers, from the perspective of the effectiveness of the advanced practice nurse, seem to indicate that APN-CCWs are beneficial. These studies are necessary to serve as a basis for subsequent evaluations comparing our country and other countries where the APN-CCW program has been implemented.

However, with the current data, the APN-CCW becomes a reference for clinical nursing staff. With its consulting, teaching, and leading work in the care of venous lesions, it allows for improvement in the implementation of preventive measures, especially compressive bandages, optimizing resources, and being available to resolve doubts that may arise in the daily care of patients. This improves the confidence and safety of professionals and patients.

## 5. Conclusions

The APN-CCW was shown to be an effective figure that promotes and improves the care of venous ulcers in the districts where it has been established.

After the intervention, the clinical outcome of patients with venous ulcers improved, including increased healing rates, decreased infection, and improved perilesional skin protection.

There was a considerable increase in compression therapy and the optimization of multilayer compression therapy, which helped to improve cure rates.

## Figures and Tables

**Table 1:** Characteristics of venous ulcers.

Venous Ulcers	Category	2015 Percentage	2016 Percentage
Depth	Bone	1.06%	1.58%
	Muscle	3.19%	6.34%
	Subcutaneous tissue	37.23%	23.80%
	Epidermis	42.55%	48.41%
	Intact skin	15.95%	25.39%
Type of tissue	Necrotic tissue	3.1%	1.58%
	Slough	22.34%	6.34%
	Granulation	42.5%	23.80%
	Epithelialization	21.2%	48.41%
	Closed	10.63%	21.42%
Edges	Thickened	9.57%	7.14%
	Damaged	35.10%	5.55%
	Delimited	32.97%	26.19%
	Diffuse	13.82%	32.53%
	Undistinguishable	2.12%	19.04%
Exudate	Leakage of exudate	5.31%	2.38%
	Saturated	13.82%	3.17%
	Wet	33.33%	18.25%
	Damp	18.08%	32.63%
	Dry	13.82%	26.19%

**Table 2:** Clinical signs of infection.

Clinical Signs of Infection	2015 Percentage	2016 Percentage	Difference
Increase of size	8.51%	6.34%	−2.17%
Increase of pain	19.14%	11.90%	−7.24%
Increase of exudate	17.02%	9.52%	−7.5%
Increase of temperature	3.19%	2.38%	−0.81%
Biofilm	3.19%	1.03%	−2.16%
Erythema	15.95%	13.49%	−2.46%
Exudate	15.95%	6.34%	−9.61%
Stagnant wound	13.82%	10.31%	−3.51%
Hypergranulation	13.82%	1.58%	−12.24%
Satellite injuries	3.19%	2.38%	−0.81%
Increased odor	4.25%	1.58%	−2.67%
Pallor	2.12%	0.79%	−1.33%
Friable tissue	15.95%	3.17%	−12.78%
RESVECH	--	12.05%	--
Fistulas	11.06%	2.38%	−8.68%

**Table 3:** Types of bandages.

Types of Bandages	2015		2016		
	*n*	%	*n*	%	**Difference**
Elastic bandage	86	52.44%	178	65.68%	+13.24%
Inelastic bandage	7	4.26%	8	2.95%	−1.30%
Multilayer bandaging	10	6,10%	59	21.77%	+15.67%
No bandage	61	37.20%	26	9.59%	−27.61%
